# Metatranscriptomic and metabolite profiling reveals vertical heterogeneity within a *Zygnema* green algal mat from Svalbard (High Arctic)

**DOI:** 10.1111/1462-2920.14788

**Published:** 2019-09-11

**Authors:** Martin Rippin, Martina Pichrtová, Erwann Arc, Ilse Kranner, Burkhard Becker, Andreas Holzinger

**Affiliations:** ^1^ University of Cologne Botanical Institute Cologne Germany; ^2^ Department of Botany University of Innsbruck Innsbruck Austria; ^3^ Department of Botany Charles University Prague Czech Republic

## Abstract

Within streptophyte green algae Zygnematophyceae are the sister group to the land plants that inherited several traits conferring stress protection. *Zygnema* sp., a mat‐forming alga thriving in extreme habitats, was collected from a field site in Svalbard, where the bottom layers are protected by the top layers. The two layers were investigated by a metatranscriptomic approach and GC–MS‐based metabolite profiling. In the top layer, 6569 genes were significantly upregulated and 149 were downregulated. Upregulated genes coded for components of the photosynthetic apparatus, chlorophyll synthesis, early light‐inducible proteins, cell wall and carbohydrate metabolism, including starch‐degrading enzymes. An increase in maltose in the top layer and degraded starch grains at the ultrastructural levels corroborated these findings. Genes involved in amino acid, redox metabolism and DNA repair were upregulated. A total of 29 differentially accumulated metabolites (out of 173 identified ones) confirmed higher metabolic turnover in the top layer. For several of these metabolites, differential accumulation matched the transcriptional changes of enzymes involved in associated pathways. In summary, the findings support the hypothesis that in a *Zygnema* mat the top layer shields the bottom layers from abiotic stress factors such as excessive irradiation.

## Introduction

Algae of the genus *Zygnema* are commonly found in Polar ecosystems, forming extensive mats in shallow pools, meltwater streams or on moist soil surfaces (Kim *et al*., 2008; Zidarova, 2008; Holzinger *et al*., 2009; Pichrtová *et al*., 2018). These habitats are characterized by extreme environmental conditions, including high seasonality. *Zygnema* spp. experience dry periods and freezing events, requiring a capability to quickly adapt to changing conditions and also abiotic stress factors in the polar climate, such as nutrient limitation and high continuous irradiance during summer (McLean and Pessoney, 1971; Hessen, 2007; Thomas *et al*., 2008). The ability of *Zygnema* sp. to form different specialized cell types such as parthenospores, akinetes and so‐called *pre‐akinetes*, which are modified vegetative cells, supports survival under unfavourable conditions (McLean and Pessoney, 1971; Stancheva *et al*., 2012; Herburger *et al*., 2015). This is especially important in polar environments, as the formation of diploid zygospores is an extremely rare event in Zygnematophyceae from Arctic regions (Elster *et al*., 1997; Pichrtová *et al*., 2018). In addition, *Zygnema* uses diverse strategies to protect itself from high levels of irradiation, both ultraviolet radiation (UVR) and photosynthetically active radiation (PAR) (Holzinger *et al*., 2009; Pichrtová *et al*., 2013; Pierangelini *et al*., 2017). Pichrtová *et al*. (2013) and Holzinger *et al*. (2018) found that *Zygnema* filaments accumulate phenolic compounds when exposed to UVR. Moreover, the surface layer in a mat may act as a ‘sunshade’ that protects the inner layers from excessive radiation (Holzinger *et al*., 2009; Karsten and Holzinger, 2014). This photoprotective mechanism has also been described for the streptophyte soil alga *Klebsormidium crenulatum* and the marine chlorophyte, *Ulva* sp. (Bischof *et al*., 2002; Karsten *et al*., 2010). The top layer of *Ulva* canopies is often completely bleached, acting as a selective UVR filter for the subcanopy thalli (Bischof *et al*., 2002). In contrast, the top layers of a *Zygnema* mat do not exhibit substantial bleaching when dried out, and although some cells die, most of the population is subsequently converted into vegetative pre‐akinetes (Holzinger *et al*., 2009).

Elevated levels of PAR and UVR can increase the production of reactive oxygen species (ROS), incurring damage to key macromolecules, such as proteins, lipids and DNA, photobleaching and photodestruction (Cockell and Knowland, 1999; Asada, 2006). Algae and plants developed protection mechanisms to diminish these effects (Asada, 2006). For example, non‐photochemical quenching (NPQ) involves quenching of potentially harmful singlet excited chlorophylls and dissipation of excess energy as heat. NPQ was detected in *Zygnema circumcarinatum*, but other streptophytes like *Klebsormidium* showed a higher NPQ (Pierangelini *et al*., 2017). Free chlorophyll molecules may be transiently bound by early light‐induced proteins (ELIPs) that protect the thylakoids from photooxidative damage and serve as sinks for excitation energy (Heddad *et al*., 2012). Potential damage by high UVR loads to DNA is counteracted by repair mechanisms, such as photoreactivation and excision repair to restore the integrity of the double helix (Cockell and Knowland, 1999; Morales‐Ruiz *et al*., 2018). For instance, Rippin *et al*. (2017) observed that vegetative filaments of *Z*. *circumcarinatum* responded to desiccation stress with a strong upregulation of a Nijmegen breakage syndrome 1 protein homologue. This protein was also found in higher plants and is involved in DNA recombination and damage repair (Akutsu *et al*., 2007). Integrity of DNA and other biomolecules is also negatively affected by the formation of ROS during high light stress, although ROS are also essential parts of important signalling pathways (Asada, 2006; Cruz de Carvalho, 2008). To counteract the accumulation of ROS, algae, as other aerobic organisms, possess a complex scavenging machinery (Maughan *et al*., 2010; Erickson *et al*., 2015). ROS‐processing enzymes such as superoxide dismutase (SOD), peroxidases, (mono‐) dehydroascorbate reductase, glutathione S‐transferase (GST), peroxiredoxins and catalases are present in the chloroplast and other cellular compartments where they scavenge ROS (Rezaei *et al*., 2013). Homologues of these proteins were identified in the desiccation stress transcriptome of *Z*. *circumcarinatum* (Rippin *et al*., 2017). These enzymes complement, or work in synergy with, low‐molecular‐weight antioxidants such as ascorbate, glutathione and tocochromanols (Kranner *et al*., 2010). In addition, carbohydrates and sugar alcohols may also scavenge ROS in photosynthetic organisms (Pommerrenig *et al*., 2018).

The streptophyte *Zygnema* is also fascinating from an evolutionary point of view, because the Zygnematophyceae were shown to be the sister lineage to the land plants (Wodniok *et al*., 2011; Wickett *et al*., 2014; De Vries and Archibald, 2018). About 700 million years ago, the Chloroplastida split into two clades, the Chlorophyta and the Charophyta (‘streptophytic green algae’), both of which contain taxa that exhibit adaptations to the terrestrial habitat. Streptophytic algae ultimately gave rise to the Embryophyta, initiating the colonization of land and the development of terrestrial ecosystems (Becker and Marin, 2009; Leliaert *et al*., 2012; Becker, 2013). It is well established that Chlorophyta and Charophyta have different strategies to prevent ROS formation in the photosynthetic apparatus. In Embryophytes and advanced streptophytic green algae (Charaphyceae, Coloechaetohyceace, Zygnematophytae), the photosystem (PS) II subunit PsbS is the main sensor. In chlorophytes and basal streptophytes, (light‐harvesting complex)‐like protein LHCSR is the key player (Gerotto and Morosinotto, 2013). Several other evolutionary innovations that accompanied the transition from basal streptophytes to advanced streptophytes are receiving increasing attention, for instance the occurrence of a *phragmoplast* (Leliaert *et al*., 2012; Nishiyama *et al*., 2018; Rensing, 2018). Rensing (2018) recently suggested that the transfer of certain plastid‐encoded genes to the nucleus in combination with other molecular changes in the last common ancestor of the Zygnematophyceae and the Embryophyta may have been key to facilitating the evolution of the land plants. Holzinger and Pichrtová (2016) suggested that the transition from aquatic to terrestrial life may have been supported by the spatial organization within algal mats and soil crusts, whereby individuals in the uppermost layers protect the layers underneath from abiotic stress factors such as desiccation and high irradiation including UVR (e.g. Graham *et al*., 1995; Berry and Lembi, 2000).

Here, we present a combined approach of metatranscriptomics and metabolite profiling in environmental samples of *Zygnema* sp. collected from the Arctic Svalbard to understand the spatial organization of *Zygnema* mats in a meltwater streamlet in which the upper layers are already exposed to higher PAR and UVR. Macroscopically distinct filaments were sampled from the top layers and the bottom layers of the same mat to assess their differences in gene expression and metabolic profile.

## Results

### *Morphological, ultrastructural and physiological characterization*

**Figure 1:**
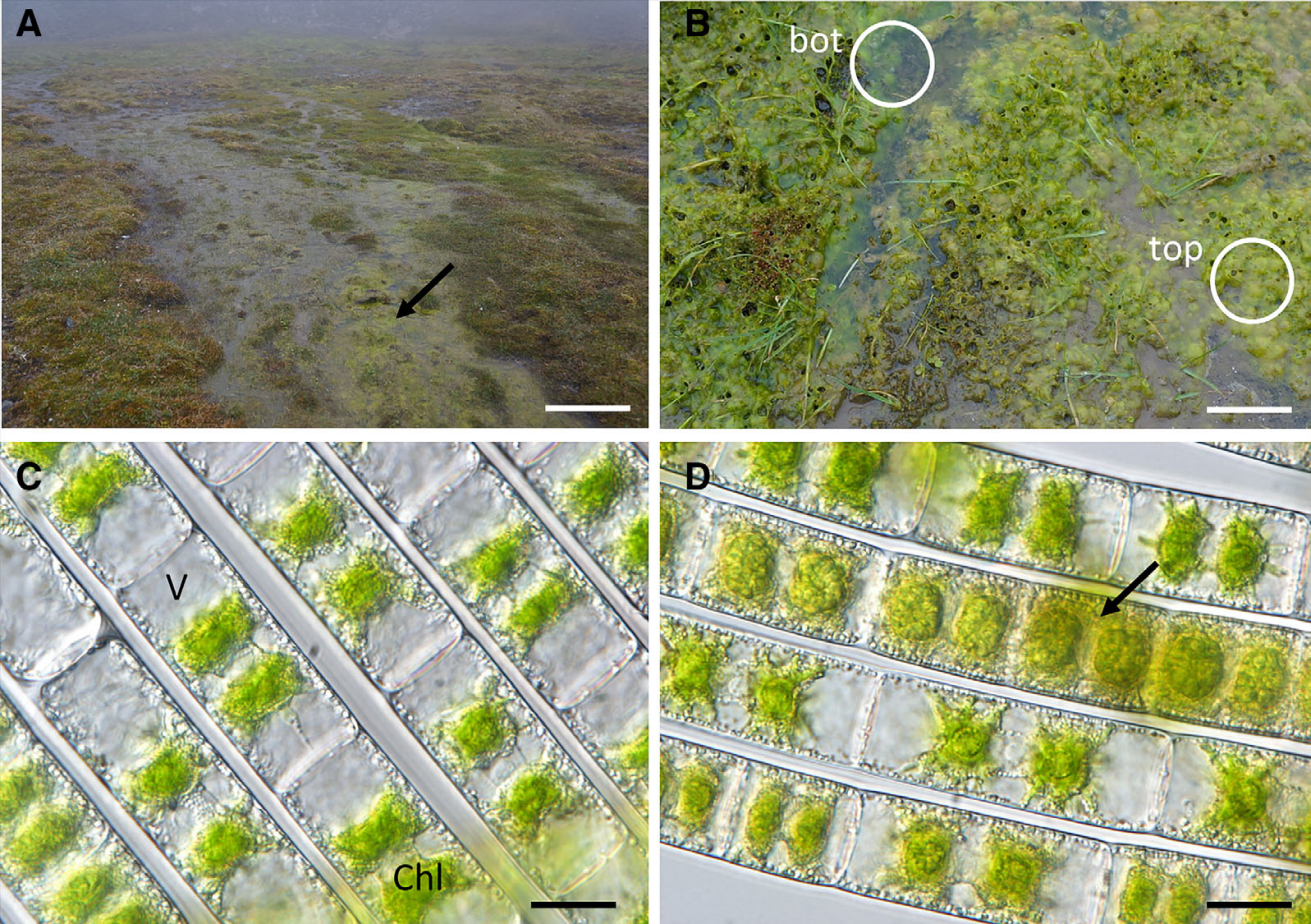
Sampling location at a plateau near Longyearbyen. A. Streamlet with dense algal population is visible in the front (arrow). B. Closer view of the algal mat with dark green cells in the submerged bottom layer (‘bot’), and pale green biomass in the top layer (‘top’, both marked with cycles). C. Young filaments of *Zygnema* sp. V with large vacuoles (V) and bright green chloroplasts from the bottom layer. D. Cells in the top layer (arrow) with yellowish content and denser appearance. Scale bars A: 2 m, B: 20 cm, C–D: 20 μm. [Color figure can be viewed at http://wileyonlinelibrary.com]

**Figure 2:**
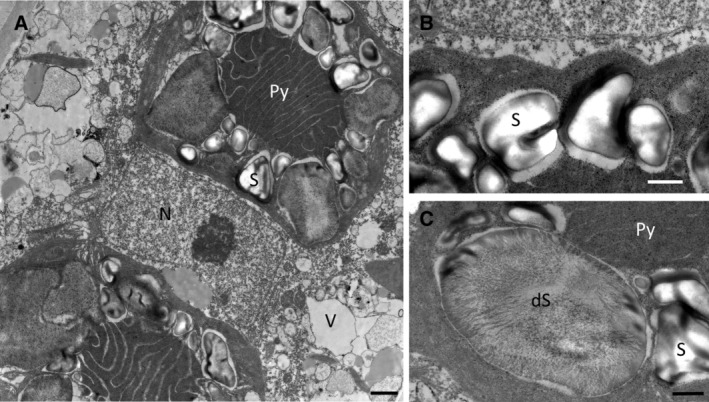
Transmission electron micrographs of *Zygnema* sp. V, young vegetative cells. A. Overview with nucleus (N) in the cell center, chloroplasts with prominent pyrenoids (Py) surrounded by starch grains (S), (B) intact starch grains typical for a sample form the bottom layer. C. Degraded starch grain (dS) with fibrillous content typical for cell of the top layer. Scale bars A: 1 μm; B–C: 0.5 μm.

The submerged dark green mats (bottom layer) contained almost exclusively freshly divided cells with two stellate chloroplasts containing large vacuoles, whereas individual filaments in the top layer were pale and contained more storage compounds (top layer, Fig. 1A–C). These cells can be regarded as vegetative cells rather than pre‐akinetes, as the latter would contain much more storage compounds (Pichrtová *et al*., 2014a). The cells in the top layer were morphologically more heterogeneous than in bottom and central layers of the mat (Fig. 1D). Transmission electron microscopy showed that vegetative cells of both layers had a central nucleus and chloroplasts with massive pyrenoids surrounded by starch grains (Fig. 2A). The starch grains of the cells collected from the bottom layer had a typical appearance with electron dense and electron translucent areas (Fig. 2B), whereas the starch grains surrounding the pyrenoids from the top layer of the mat had an irregular shape and the central contents appeared fibrillosus, which can be regarded as a degradation product (Fig. 2C). Relative electron transport rates (rETR) measurements showed slightly different kinetics (light compensation points *I*_k_ for the bottom layer 156.3 and for the top layer 91.5 μmol photons m^−2^ s^−1^) between the two samples, mean rETR_max_ values of the bottom layer were higher than for the top layer (Fig. S1).

### *Algal mat composition*

**Figure 3:**
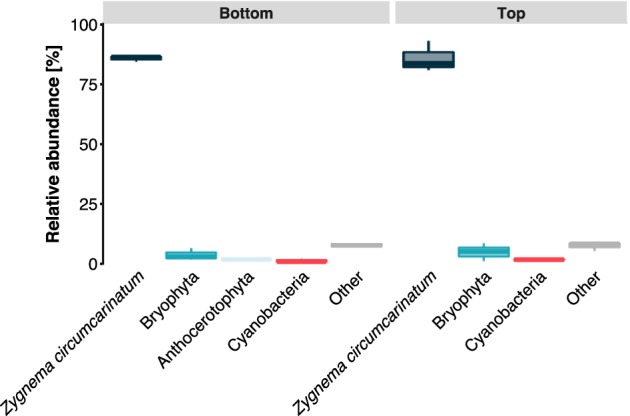
Taxonomic relative abundances in the two investigated layers of a mat. Reads, obtained from bottom layer (left) and top layer (right), could be mapped to the small subunit of the rRNA gene of either *Zygnema circumcarinatum*, Bryophyta, Anthocerotophyta, cyanobacteria or other (<0.1%). [Color figure can be viewed at http://wileyonlinelibrary.com]

The filtered 16S and 18S rRNA gene sequences (see below) were analysed and the results indicated a high relative abundance (>85%) of *Z*. *circumcarinatum* (Fig. 3). Additionally, some reads belonging to Bryophyta, Anthocerotophyta, cyanobacteria and minor counts of other organisms were detected. Cytochrome *c* oxidase subunit I of a mosquito belonging to the genus *Aedes* was found in small proportions in the transcripts. The mat can be clearly described as *Zygnema* dominated, and the strain investigated in this study has been previously characterized as *Zygnema* sp. V by *rbc*L phylogeny (Pichrtová *et al*., 2018).

### *Metatranscriptomic analysis*

The sequencing runs produced 26 845 Mbp for the reference library and 98 462 Mbp in total for the sample libraries. The quality‐filtered reads of the reference library were assembled to 122 089 transcripts with an N50 of 764 bp and a total base count of 79 578 347.

**Figure 4:**
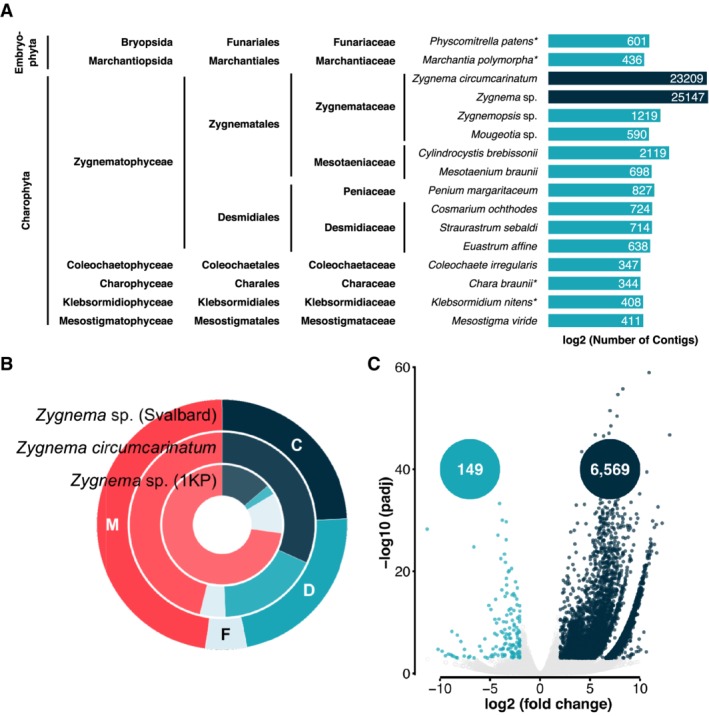
Results of the metatranscriptomic analysis. A. BLASTN search against several Streptophyta using the metatranscriptome as a query. The bars, representing the number of hits, were log2 transformed; however, the numbers are the actual numbers of contigs that generated a hit. B. BUSCO analysis results for the *Zygnema* sp. (Svalbard) metatranscriptome, analysed in this study, *Zygnema circumcarinatum* (Rippin *et al*., 2017) and *Zygnema* sp. from the 1KP project. The BUSCO categories are abbreviated with C (complete; dark blue), D (duplicated; turquoise), F (fragmented; light blue) and M (missing; red). C. Volcano plot showing the results of the differential gene expression analysis. Transcripts with a fold change of at least 4 and a *p*_adj_ of less than 0.001 were considered as differentially expressed. Upregulated transcripts of the top layer are depicted in dark blue while downregulated transcripts are turquoise. [Color figure can be viewed at http://wileyonlinelibrary.com]

The assembly was analysed using BLASTN and several transcriptomes and genomes of charophytic algae and two embryophytes. Highest annotation rates were achieved for *Zygnema* sp. (1KP project) and *Z*. *circumcarinatum* (Rippin *et al*., 2017), followed by *Cylindrocystis brebissonii* and *Zygnemopsis* sp. (Fig. 4A). The lowest overlap was found for *Coleochaete irregularis* and *Chara braunii*. The annotation rate against the SWISSPROT database was 28.3% of all transcripts (Table S1).

The benchmarking single copy orthologues (BUSCO) analysis of the assembled sequences found 46.8% of the orthologues to be complete, 5.3% to be fragmented and 47.9% missing (Fig. 4B). Thus, the assembly is in a similar range as the transcriptome of *Zygnema circumcarinatum* and showed a higher coverage of orthologues than the transcriptome of *Zygnema* sp. of the 1KP project. Kyoto Encyclopaedia of Genes and Genomes (KEGG) orthology (KO) terms were assigned to the transcripts and subsequently mapped onto the KEGG metabolic pathway map (ko01100). The major pathways, such as carbohydrate metabolism, amino acid metabolism, fatty acid metabolism, nucleotide metabolism and respiration, were well covered, confirming the high quality of the assembled transcriptome (Fig. S2).

**Figure 5:**
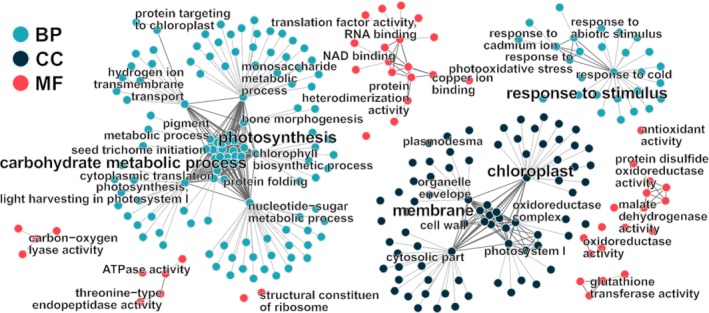
GO network plot representing all terms enriched in the upregulated fraction of the metatranscriptome. The GO root categories are abbreviated with BP (biological process; turquoise), CC (cellular component; dark blue) and MF (molecular function; red). [Color figure can be viewed at http://wileyonlinelibrary.com]

Figure 4C shows a volcano plot of differentially expressed genes in top layer compared with the bottom layer. In the top layer, a total of 6569 genes were significantly upregulated, whereas only 149 were downregulated. A functional overview of the regulated genes was obtained by means of gene ontology (GO) and KEGG enrichment analyses. The GO analysis revealed an enrichment of 270 terms (145 biological processes, 78 cellular components, 47 molecular functions) in the upregulated fraction and 16 (seven biological processes, one cellular component, eight molecular functions) for the downregulated genes (Table S2). The 270 GO terms associated with the induced transcripts were clustered according to their relationships and plotted as a network in Fig. 5. A high number of categories were related to photosynthesis, carbohydrate metabolism, transcription and translation as well as stress response. Table 1 shows the results of the KEGG enrichment analysis for the upregulated transcripts. The ath (*A*. *thaliana*) pathways for ribosome, oxidative phosphorylation, carbon metabolism, proteasome, protein processing in endoplasmic reticulum, carbon fixation in photosynthetic organisms, pyruvate metabolism, citrate cycle and phagosome showed upregulation. Downregulated pathways were not observed.

**Table 1:** Result of the KEGG enrichment analysis.

Pathway ID	Pathway name	*p*_value_	*p*_adj_
ath03010	Ribosome	8.01E‐07	6.72E‐05
ath00190	Oxidative phosphorylation	5.63E‐06	2.36E‐04
ath01200	Carbon metabolism	1.06E‐05	2.96E‐04
ath03050	Proteasome	5.17E‐05	1.09E‐03
ath04141	Protein processing in endoplasmic reticulum	7.43E‐05	1.25E‐03
ath00710	Carbon fixation in photosynthetic organisms	1.59E‐04	2.23E‐03
ath00620	Pyruvate metabolism	3.89E‐04	4.67E‐03
ath00020	Citrate cycle (TCA cycle)	1.39E‐03	1.30E‐02
ath04145	Phagosome	1.39E‐03	1.30E‐02

Table S3 shows all upregulated and downregulated transcripts and the annotation results for SWISSPROT. For better overview, a selected number of genes were categorized (Table 2). Several components of PS I and II, light‐harvesting complexes and the cytochrome b6‐f complex were upregulated alongside transcripts involved in chlorophyll metabolism, such as chlorophyllide *a* oxygenase, chlorophyll synthase and ELIPs. Enhanced transcription of gene products involved in carbohydrate metabolism including starch degrading enzymes, e.g. ɑ‐amylase, isoamylase and 4‐alpha‐glucanotransferase, and trehalose‐phosphate phosphatase. Amino acid metabolism and cell wall biosynthesis were upregulated in the top layer when compared with the bottom layer of the mat. The top layer of the *Zygnema* mat showed elevated transcript levels associated with carotenoid and vitamin B_6_ biosynthesis, ascorbate and thioredoxin metabolism, and other ROS scavengers. Other stress‐related transcripts, such as chaperones and heat shock proteins (Hsps), and genes involved in DNA repair were also upregulated in the top layers. In summary, photosynthesis, carbohydrate and amino acid metabolism, cell wall biosynthesis and antioxidant protection were upregulated in the top layer compared with the bottom layer.

**Table 2:** Selected differentially expressed transcripts belonging to the categories photosynthesis, chlorophyll metabolism, carbohydrate metabolism, amino acid metabolism, cell wall modifications, antioxidant defence and chaperones and DNA repair.

Transcript ID	SWISSPROT annotation	*E*_value_	Fold change	*p*_value_	*p*_adj_
Photosynthesis					
TR38390|c0_g1	Photosystem I reaction center subunit II, chloroplastic	2.94E‐90	5.55	5.46E‐21	9.28E‐19
TR89209|c3_g5	Photosystem I reaction center subunit III, chloroplastic	4.68E‐75	4.01	1.49E‐12	8.58E‐11
TR17303|c3_g1	Photosystem I reaction center subunit XI, chloroplastic	2.04E‐63	3.46	9.38E‐11	3.98E‐09
TR90674|c0_g5	Photosystem II 10 kDa polypeptide, chloroplastic	1.75E‐25	6.25	2.23E‐35	2.44E‐32
TR29264|c0_g1	Photosystem II 22 kDa protein, chloroplastic	9.04E‐75	7.56	6.57E‐31	3.43E‐28
TR31924|c0_g1	Photosystem II stability/assembly factor HCF136, chloroplastic	8.25E‐170	7.45	1.44E‐21	2.64E‐19
TR15792|c2_g2	Chlorophyll a‐b binding protein type 1 member F3, chloroplastic	1.67E‐120	6.89	6.35E‐33	4.38E‐30
TR27873|c0_g7	Chlorophyll a‐b binding protein, chloroplastic	1.08E‐127	7.24	7.31E‐36	8.35E‐33
TR76752|c0_g1	Chlorophyll a‐b binding protein, chloroplastic	3.41E‐117	6.10	7.13E‐41	1.63E‐37
TR90376|c0_g2	Cytochrome b6‐f complex subunit 4	2.28E‐107	3.74	1.30E‐06	2.38E‐05
TR95287|c0_g2	Early light‐induced protein 1, chloroplastic	7.79E‐31	8.73	5.74E‐12	3.02E‐10
TR37248|c0_g3	Early light‐induced protein 2, chloroplastic	9.57E‐23	2.54	1.27E‐06	2.34E‐05
Chlorophyll metabolism					
TR81529|c0_g2	Chlorophyll synthase, chloroplastic	2.26E‐165	7.70	5.63E‐15	4.56E‐13
TR36941|c0_g2	Chlorophyllase‐2, chloroplastic	1.48E‐69	5.28	9.40E‐07	1.80E‐05
TR12670|c0_g1	Chlorophyllide a oxygenase, chloroplastic	1.31E‐146	4.55	5.57E‐06	8.74E‐05
TR58878|c0_g1	Geranylgeranyl diphosphate reductase, chloroplastic	4.59E‐146	3.30	1.89E‐06	3.33E‐05
TR52618|c1_g2	Magnesium‐chelatase subunit ChlD	0	7.90	1.60E‐22	3.30E‐20
Carbohydrate metabolism					
TR57182|c0_g1	alpha‐Amylase 2	1.44E‐58	8.02	4.56E‐08	1.18E‐06
TR90623|c0_g1	Isoamylase 1, chloroplastic	0	5.81	2.26E‐08	6.22E‐07
TR4678|c0_g1	4‐alpha‐Glucanotransferase, chloroplastic/amyloplastic	3.83E‐136	7.68	1.26E‐07	2.95E‐06
TR48517|c0_g1	Trehalose‐phosphate phosphatase B	4.15E‐57	7.24	4.38E‐06	7.07E‐05
Amino acid metabolism					
TR74126|c0_g1	Alanine aminotransferase 2	9.38E‐101	8.27	1.38E‐08	3.95E‐07
TR30432|c0_g1	D‐3‐phosphoglycerate dehydrogenase 1, chloroplastic	0	6.82	5.22E‐21	8.92E‐19
TR87246|c1_g2	Dihydrolipoyl dehydrogenase 1, mitochondrial	0	6.15	2.41E‐19	3.45E‐17
TR67795|c0_g3	Glycine dehydrogenase (decarboxylating), mitochondrial	0	3.05	1.23E‐05	0.000177487
TR17239|c1_g2	Serine hydroxymethyltransferase, mitochondrial	0	5.66	1.19E‐16	1.22E‐14
TR15202|c1_g1	Tryptophan synthase alpha chain	1.44E‐98	6.21	2.35E‐09	7.82E‐08
TR51269|c0_g1	Tryptophan synthase beta chain 2, chloroplastic	0	4.54	1.02E‐18	1.34E‐16
Cell wall modifications					
TR20263|c0_g1	Expansin‐A9	1.42E‐54	6.33	1.99E‐17	2.25E‐15
TR90351|c0_g1	Expansin‐A30	8.18E‐51	7.36	1.41E‐25	3.89E‐23
TR70445|c0_g1	Xyloglucan 6‐xylosyltransferase 3	2.12E‐135	6.46	2.06E‐12	1.16E‐10
TR76780|c0_g1	Xyloglucan endotransglucosylase/hydrolase protein 22	3.01E‐34	4.11	1.29E‐07	3.02E‐06
Antioxidant defence					
TR69233|c0_g2	beta‐Carotene 3‐hydroxylase 1, chloroplastic	9.58E‐59	4.64	1.26E‐05	0.000182169
TR58091|c0_g1	Carotene epsilon‐monooxygenase, chloroplastic	6.75E‐120	7.50	7.97E‐07	1.55E‐05
TR9069|c0_g1	Lycopene epsilon cyclase, chloroplastic	5.95E‐57	8.01	1.71E‐08	4.82E‐07
TR43663|c0_g1	Phytoene dehydrogenase	2.59E‐21	5.29	9.63E‐07	1.84E‐05
TR87265|c0_g3	Phytoene synthase, chloroplastic	1.35E‐161	7.91	4.75E‐22	9.29E‐20
TR62830|c0_g1	zeta‐Carotene desaturase, chloroplastic/chromoplastic	4.02E‐69	7.38	2.19E‐06	3.82E‐05
TR29247|c1_g1	L‐ascorbate peroxidase 2, cytosolic	4.60E‐112	6.38	1.67E‐29	7.66E‐27
TR76496|c0_g1	Mono‐dehydroascorbate reductase	0	6.60	1.91E‐24	4.72E‐22
TR51268|c0_g1	Glutathione reductase, chloroplastic/mitochondrial	0	3.95	7.20E‐13	4.35E‐11
TR26660|c0_g1	Thioredoxin H‐type	3.58E‐29	7.11	4.45E‐15	3.68E‐13
TR43732|c0_g2	Thioredoxin M‐type, chloroplastic	4.45E‐49	9.74	1.80E‐15	1.56E‐13
TR46713|c0_g1	Thioredoxin reductase NTRC	0	6.09	3.89E‐11	1.77E‐09
TR8093|c0_g1	Pyridoxal 5′‐phosphate synthase subunit PDX1	4.00E‐160	4.63	1.06E‐15	9.51E‐14
TR8631|c0_g2	Pyridoxal reductase, chloroplastic	4.00E‐150	8.36	1.07E‐08	3.15E‐07
TR49658|c0_g1	Catalase	4.16E‐82	6.46	5.63E‐13	3.47E‐11
TR30511|c0_g1	Copper chaperone for superoxide dismutase, chloroplastic/cytosolic	3.09E‐100	8.57	1.60E‐10	6.48E‐09
TR8666|c0_g1	Superoxide dismutase [Cu‐Zn], chloroplastic	4.09E‐73	7.66	1.74E‐07	3.94E‐06
TR82265|c0_g1	Superoxide dismutase [Fe] 2, chloroplastic	2.61E‐51	7.04	1.50E‐05	0.000211418
TR8986|c0_g2	Superoxide dismutase [Mn] 1, mitochondrial	1.04E‐97	8.62	4.80E‐11	2.15E‐09
TR90325|c0_g1	Glutathione S‐transferase F10	5.96E‐69	6.05	3.20E‐19	4.51E‐17
TR74319|c0_g1	Microsomal glutathione S‐transferase 3	8.26E‐30	9.12	2.89E‐13	1.86E‐11
TR30448|c0_g1	Peptide methionine sulfoxide reductase	1.71E‐71	5.03	8.77E‐07	1.69E‐05
TR42383|c0_g1	Peroxiredoxin Q, chloroplastic	1.31E‐70	7.25	1.82E‐21	3.30E‐19
Chaperones and DNA repair					
TR87681|c0_g1	18.5 kDa class I heat shock protein	7.42E‐37	10.90	1.48E‐64	1.19E‐59
TR51363|c0_g2	Chaperone protein ClpB1	7.45E‐103	6.62	8.69E‐05	0.000993204
TR37257|c0_g1	Chaperone protein DnaJ	4.41E‐98	6.74	2.03E‐10	8.12E‐09
TR10870|c0_g1	Heat shock 70 kDa protein, mitochondrial	0	6.80	4.05E‐05	0.00051142
TR60558|c0_g2	Cullin‐4	1.01E‐131	8.01	1.93E‐07	4.33E‐06
TR18936|c0_g1	DNA damage repair/toleration protein DRT100	1.85E‐14	7.67	4.60E‐07	9.49E‐06
TR73829|c0_g1	DNA repair protein RAD51 homologue 1	2.00E‐121	7.13	7.54E‐06	0.000114517
TR19814|c0_g1	RING‐box protein 1a	1.79E‐59	8.46	1.30E‐08	3.75E‐07

### *Metabolite profile*

**Figure 6:**
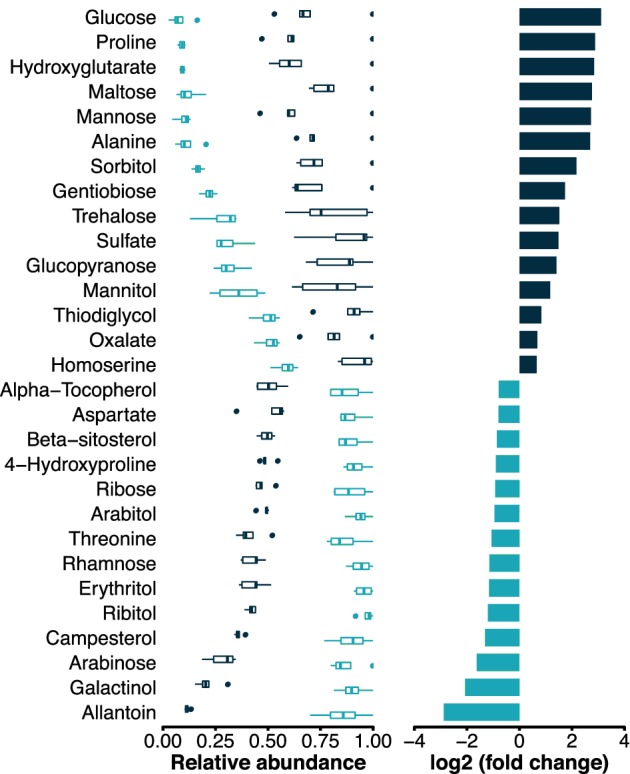
Differential accumulation analysis of the metabolite profile. The relative abundance of the metabolites is given on the left in turquoise (bottom layer) and dark blue (top layer). In the right panel, the fold change from the bottom layer to the top layer is displayed where dark blue represents differentially accumulated compounds and turquoise differentially depleted. The threshold was set to a fold change of 1.5 and *p*_adj_ of less than 0.01. The complete results are included in Table S4. [Color figure can be viewed at http://wileyonlinelibrary.com]

GC–MS based metabolite profiling was used to further investigate the composition of the different layers of the mat. Out of a total of 173 detected molecules, mostly comprising primary metabolites, 43 exhibited a differential accumulation or depletion (Table S4). Annotations were available for 29 of these compounds of which 15 were accumulated in the top layer, and 14 were accumulated in the bottom layer (Fig. 6). In the top layer, glucose, maltose, mannose and sorbitol were the most up‐accumulated sugars and sugar alcohols. Proline and alanine were the most up‐accumulated amino acids. In contrast, galactinol, arabinose and campesterol were the most depleted sugars and sugar alcohols from the top layer. Allantoin, a degradation product of nucleic acids was also strongly depleted in the top layer.

## Discussion

We analysed the top and the bottom layers of an algal mat, dominated by *Zygnema* sp., from Svalbard, Norway. As all samples for the present study were collected from one field site, the investigated species was previously characterized by *rbc*L sequences as *Zygnema* sp. V, belonging to the *Z*. *circumcarinatum* clade (Pichrtová *et al*., 2018). The mat was sampled in early August during the Arctic growing season when water is readily available from melting snowfields. August is also the period of the midnight sun with constant exposure of organisms to high irradiation (Digby, 1960; Arendt, 2012). As the mats are fully exposed to sun light, the top layers of such algal mats shade the bottom layers and protect them from light stress (Holzinger *et al*., 2009; Karsten and Holzinger, 2014); we interpret this as a general strategy where all individual cells have a benefit. Our metatranscriptomic data of the *Zygnema* sp. mat clearly showed that the top layer had a higher metabolic turnover compared with the cells from the bottom layer and exhibited acclimation to light and UVR, and in general stress protection. The induction of stress‐related transcript changes observed here are very similar to observations in the charophytes *Coleochaete* and *Spirogyra* (Timme and Delwiche, 2010), the moss *Physcomitrella* (Khraiwesh *et al*., 2015), pointing out the evolutionary significance of our findings.

### *Photosynthesis*

The higher ETR_max_ of bottom layers compared with the top layers (Fig. S1) indicates that the filaments in the top layer of the *Zygnema* mat protected those in the lower layer, in agreement with the need of photoautotrophic organisms to acclimate and adapt to varying light conditions (Erickson *et al*., 2015). It has been reported repeatedly for *Zygnema* sp. that higher irradiation including UV‐B may lead to a decrease in rETR values (Holzinger *et al*., 2009, 2018; Herburger *et al*., 2015) as also known from other Zygnematophyceae such as *Cosmarium* (Stamenkovic and Hanelt, 2014). The marine chlorophyte, *Ulva* sp., showed a similar trend with rETR curves of four different layers ordered reversely to their position in the stack (Bischof *et al*., 2002).

At the transcriptome level, the top layer of the *Zygnema* mat showed a higher expression of photosynthetic genes than the lower layer. Similar observations have been made after high light treatment (600 μmol photons m^−2^ s^−1^) in various Charophytes including *Zygnema* sp. (De Vries and Archibald, 2018). Particularly *Z*. *circumcarinatum* devotes a larger transcriptional effort to plastid targeted proteins (De Vries and Archibald, 2018). The same holds true for the filaments of the top layer of the *Zygnema* mat in the present study suggesting that the alga continuously replaces damaged components of the PSs. Additionally, *Zygnema* induced the expression of photoprotective proteins ELIP 1 and 2 in the top layer. These proteins belong to the chlorophyll *a*/*b*‐binding superfamily and are mainly responsive to light and UVR stress (Hayami *et al*., 2015). In addition, in the Zygnematophyceae *Spirogyra* sp. ELIPs are strongly regulated upon cold stress (Han and Kim, 2013). The chlorophyte *Chlamydomonas reinhardtii* showed a similar trend when exposed to high light (Teramoto *et al*., 2004). ELIPs are also upregulated in *Z*. *circumcarinatum* in response to desiccation treatment confirming that these proteins are generally involved in responses to abiotic stress factors (Rippin *et al*., 2017).

### *Carbohydrate metabolism*

Starch is an important energy storage in algae, which also provides carbon skeletons for other molecules and reductants (Mitsue León‐Saiki *et al*., 2017). The expression profile of the top layer of the *Zygnema* mat showed an upregulation of several enzymes involved in starch degradation. Metabolite profiling revealed an accumulation of maltose and glucose, providing further evidence for starch catabolism and higher metabolic turnover in the filaments in the top layer. The increased transcript levels of the trehalose phosphate phosphatase were congruent with an accumulation of trehalose in the metabolite profile. These non‐reducing disaccharides generally accumulate in response to different abiotic stressors to protect proteins and membranes from denaturation (Elbein *et al*., 2003; Fernandez *et al*., 2010; Lunn *et al*., 2014). In *Zygnema*, these findings could point towards beginning acclimation to desiccation, when the upper layers are in direct contact with air and experience mild desiccation stress (Pichrtová *et al*., 2014a, 2014b).

In addition, trehalose can act as a free‐radical scavenger protecting the cell from ROS (Elbein *et al*., 2003). The highly hydroxylated and soluble sugars may generally serve as efficient ROS quenchers and in membranes may be involved in scavenging hydroxyl radicals generated by lipid peroxidation. In response to abiotic stress factors, such as high light or low temperatures, higher plants typically accumulate the disaccharide sucrose as well as the monosaccharides glucose and fructose (Pommerrenig *et al*., 2018). Sugar alcohols possess more hydroxyl groups than their sugar precursors, and often accumulate in response to oxidative stress (Pommerrenig *et al*., 2018). The metabolite profile of *Zygnema* also indicated an accumulation of the sugar alcohols sorbitol and mannitol in the top layer of the mat, which could be a response to ROS formation. Arabitol and ribitol were depleted in the top layer, which could be interpreted as a shift towards sugar alcohols with more hydroxyl groups such as sorbitol and mannitol. The concentration of the pentose ribose was also decreased in the top layer compared with the bottom layer. Several other key metabolites, such as ATP, hormones, NAD and nucleotides, contain ribose or its derivatives. This sugar can also be metabolized via the pentose phosphate pathway, glycolysis and tricarboxylic acid (TCA) cycle to generate energy (Riggs *et al*., 2016). Certain sugars can also be used in cell wall biosynthesis and modification. For instance, rhamnose and arabinose are incorporated in plant cell walls in response to abiotic stress (Tenhaken, 2015). Our metabolite data showed a decrease of both sugars in the top layer of the *Zygnema* mat, providing evidence for cell wall modification.

### *Cell wall and membrane*

The cell walls of charophyte algae recently received a lot of attention, linking their composition to stress resistance and adhesion (De Vries *et al*. (2018); Holzinger and Pichrtová, 2016, Palacio‐López *et al*., 2019, Herburger *et al*., 2019). The metatranscriptome of the top layer of the *Zygnema* mat exhibited an induction of various enzymes involved in cell wall formation and modification. For example, expansins are crucial for cell wall remodelling in response to abiotic factors (Marowa *et al*., 2016). Vannerum *et al*. (2011) identified expansin homologues in the streptophyte alga *Micrasterias denticulata* and argued that these proteins have similar functions in algae as in plants. Similar to expansins, xyloglucan endotransglucosylases act on the xyloglucans of the cell wall to loosen those polymers and enable modifications (Van Sandt *et al*., 2007). Xyloglucan xylosyltransferases, on the other hand, are involved in biosynthesis of xyloglucan, one of the most abundant hemicellulosic components of the cell wall (Culbertson *et al*., 2016). In *Z*. *circumcarinatum*, the occurrence of xyloglucan:xyloglucan endotransglucosylase, for instance, was observed in young longitudinal cell walls (1 month) but not in old cells (1 year). Even novel transglycosylation activities were described between xyloglucan and xylan, xyloglucan and mannan, illustrating the importance of cell wall modifying enzymes in several charophytes including *Z*. *circumcarinatum* (Herburger *et al*., 2018). Arabinogalactan proteins have been detected in *Z*. *circumcarinatum*, possibly participating in adhesion phenomena (Palacio‐López *et al*., 2019). Cell wall modifications of the major pectin compound homogalacturonan have recently described in correlation with an increased desiccation tolerance of older cells of *Z*. *circumcarinatum* (Herburger *et al*., 2019). In 12‐month‐old cells, GalA was ~50% higher than in young cells (Herburger *et al*., 2019). The genome analysis of *C*. *braunii* showed land plant like cell wall metabolic pathways (Nishiyama *et al*., 2018), despite that this organism is strictly aquatic. Cell wall modifying enzymes leading to cell wall rigidity and imperviousness have been described to play a crucial role in early land plants hydration control, as shown by the analysis of the *Marchantia polymorpha* genome (Bowman *et al*., 2017).

The top layer of the *Zygnema* mat investigated in the present study also showed a depletion of β‐sitosterol and campesterol. Both compounds may be incorporated into biomembranes in response to environmental stress (Deng *et al*., 2016). The chlorophyte *C*. *reinhardtii* accumulates these metabolites when exposed to high light for a short period of time (Erickson *et al*., 2015).

### *Amino acid metabolism*

On the transcriptional level, amino acid metabolism was differentially regulated in the top layer of the mat. Amino acids are important building blocks for various biomolecules, especially proteins, but are also involved in other processes, such as stress signalling (Hildebrandt *et al*., 2015). Proline, for example, has multiple functions: It has osmolytic properties, protects from oxidative damage, stabilizes subcellular entities and works as a metal chelator and signalling molecule (Hayat *et al*., 2012). The metabolite analysis revealed an accumulation of proline at the top layer of the *Zygnema* mat compared with the bottom. Other amino acids, such as hydroxyproline, are essential constituents of the cell wall (Golan‐Goldhirsh *et al*., 1990). Thus, the depletion of hydroxyproline in the metabolite profile of the top layer could indicate its use in cell wall modifications. In contrast, the abundance of hydroxyglutarate increased in the top layer. Hydroxyglutarate is formed during lysine degradation and can be introduced into the TCA cycle as 2‐ketoglutarate for energy generation (Engqvist *et al*., 2014).

### *Effective antioxidant defence*

Biotic and abiotic stress factors in photosynthetic organisms frequently lead to an increased production of ROS, which has the potential to render all major biomolecules dysfunctional (Demidchik, 2015). To control ROS levels, algae and higher plants possess intra‐ and extra‐cellular antioxidant defence mechanisms involving ascorbate, glutathione, tocochromanols and other isoprenoids, flavonoids as well as enzymatic antioxidants (Demidchik, 2015). The expression of antioxidant‐based defence mechanisms was generally upregulated in *Zygnema* filaments in the top layer, suggesting that they needed better protection from ROS than those in the lower layers.

The metatranscriptomic analysis showed that the expression of a copper chaperone for SOD, several SODs, ascorbate peroxidase, mono‐dehydroascorbate reductase and glutathione reductase was upregulated. Whereas the copper chaperone for SOD delivers copper to the copper/zinc SOD, the different metalloforms of SODs act directly on ROS (Cizewski Culotta *et al*., 1997; Asada, 2006). *Zygnema* has three different organelle‐specific SODs, the manganese, iron and copper/zinc metalloforms. For instance, copper/zinc and iron SODs are located in plastids (Wolfe‐Simon *et al*., 2005).

The multifunctional tripeptide antioxidant glutathione is transferred by GSTs, conjugating it to electrophilic compounds acting as peroxidases or dehydroascorbate reductases (Noctor *et al*., 2011; Rezaei *et al*., 2013). The metatranscriptome of the *Zygnema* mat showed an upregulation of different GSTs in the top layer of the mat. The enzymes catalase, peptide methionine sulfoxide reductase (MsrA) peroxiredoxin Q, thioredoxin H and M as well as the thioredoxin reductase (TrxR) exhibited enhanced expression. Catalase and peroxiredoxin Q also participate in the degradation of hydrogen peroxide. Oxidized peroxiredoxin is subsequently reduced by the TrxR and thioredoxin (Cha *et al*., 2015). MsrA, on the other hand, is a repair enzyme, which acts on damaged proteins and catalyses the conversion of methionine sulfoxide back to methionine (Weissbach *et al*., 2002). Thiol‐disulphide conversions through the intricate network of glutathione and related proteins such as GSTs, thioredoxins and glutaredoxins, play central roles in plant response to abiotic stress factors (Zagorchev *et al*., 2013). Therefore, the here observed up‐regulation of compounds involved in antioxidant defence and thiol‐disulphide conversions in the filaments of the top layer provide further evidence for a higher oxidative challenge compared with filaments in the bottom layer. Carotenoids, isoprenoid compounds consisting of eight isoprene units, are another important group of ROS scavengers (Harjes *et al*., 2008; Havaux, 2013). Apart from the roles in photosynthesis, carotenoids are able to quench singlet oxygen and deactivate triplet states of chlorophyll (Horton and Ruban, 2005; Jahns and Holzwarth, 2012).

The top layer of the *Zygnema* mat showed an induction of the carotenoid biosynthesis, such as an upregulation of phytoene synthase, phytoene dehydrogenase, ζ‐carotene desaturase, lycopene ε‐cyclase, β‐carotene 3‐hydroxylase and carotene ε‐monooxygenase. Lutein is another photoprotective molecule that can quench triplet chlorophyll (Jahns and Holzwarth, 2012). Pyridoxine and its derivatives (the Vitamin B_6_ group) are also involved in photoprotection and minimizing oxidative damage in photosynthetic organisms (Havaux *et al*., 2009). The top layer of the *Zygnema* mat induced the pyridoxal reductase and pyridoxal 5′‐phosphate synthase, again indicating a higher need for photoprotection.

### *Chaperones and DNA repair*

Chaperones and Hsps are crucial parts of abiotic stress response as they refold misfolded proteins and protect them from aggregation (Wang *et al*., 2004; Al‐Whaibi, 2011). The upper layers of an *Ulva rotundata* mat showed increased concentrations of chaperonine 60 when exposed to light or UVR stress (Bischof *et al*., 2002). The top layer of the *Zygnema* mat also induced the expression of Hsps and chaperones, e.g. the chaperones ClpB1 and DnaJ. Desiccation stress also led to an upregulation of these two enzymes in *Z*. *circumcarinatum*, suggesting that the chaperones might generally be responsive to abiotic stress (Rippin *et al*., 2017).

High light intensities and UVR may also cause DNA lesions and cross‐linking either directly by UV A and UV B exposure or indirectly through ROS generation (Cadet and Wagner, 2013). Thus, photosynthetic organisms had to establish protection and repair mechanisms to maintain DNA integrity. It has previously been shown that *Zygnema* tolerates experimental UV A and UV B treatments very well (Holzinger *et al*., 2009; Pichrtová *et al*., 2013), and hardly any structural or metabolic changes have been observed in *Zygnema* ssp. from different origins including arctic (Holzinger *et al*., 2018). Thus, it can be concluded that the protection strategies found by the transcriptional changes in the top layers lead to a highly effective damage repair. The effectiveness of DNA repair becomes also evident from the observation that allantoin, a degradation product of nucleic acids, is strongly depleted from the top layers in the metabolite profile. De Vries *et al*. (2018) point out that *Zygnema* devotes a larger proportion of transcriptional budget to plastid‐ targeted proteins than all other investigated streptophytes, which corroborates its evolutionary significance.

### *Habitats for other microorganisms*

While we focused on the analysis of the dominating organism *Zygnema*, these mats are also microhabitats for other microorganisms. The analysis of the SSU sequence reads showed a small relative abundance of cyanobacteria frequently associated with filamentous green algae (Kim *et al*., 2008; Komárek *et al*., 2012), but this needs further corroboration as we investigated enriched mRNA. Additionally, bryophytic sequences were detected in the metatranscriptomic data set. Figure 1A shows that the sampling location was covered with bryophytes, which are the dominant vegetation cover at Svalbard (Williams *et al*., 2017). In addition, reads for cytochrome *c* oxidase from the Arctic mosquito, *Aedes* sp., were also found in the metatranscriptome. *Aedes nigripes* is commonly found in arctic regions, laying eggs in terrestrial and hydro‐terrestrial habitats (Robert *et al*., 2011). Kühlhorn (1958) observed that Culicidae larvae feed on *Zygnema*. All together, these sequences account for a small proportion of the total reads mapped to the SSU (Fig. 3) and the investigated habitat was clearly *Zygnema* dominated.

## Conclusions

The *Zygnema* mat extracted from a natural habitat at Svalbard (High Arctic) consisted of different layers. Similar to other algae, the top layer, which was transcriptionally very active (>6500 upregulated genes covering energy metabolism, photosynthesis, photoprotection and protection from oxidative stress as well as cell wall modifications), appeared to act as a sunshade for bottom layers. In addition, metabolic turnover was generally higher in the top layer. The upregulation of protection mechanisms in the top layer, likely to be an immediate response to stress while the mat is still fully submerged in water may also confer enhanced protection against future abiotic stress factors, such as desiccation, an environmental factor regularly experienced by *Zygnema* when their habitats dry out, contributing to the evolutionary success of the species.

## Experimental procedures

### *Sampling*

On August 11, 2015, samples were collected from a snowmelt‐fed streamlet at the mountain Sverdruphamaren in close vicinity to the settlement Longyearbyen, Svalbard, Norway (78°13.153′ N, 15°35.088′ E; temperature 4.7 °C; conductivity 40 μS cm^−1^; pH 7.3). Dark green filaments were collected from the fully submerged center and bottom of the mat (termed ‘bottom layer’) and light green filaments were taken from the top layer and the margins of the mat (termed ‘top layer’). Both samples contained young vegetative cells and belonged to genotype V, previously described by Pichrtová *et al*. (2018). Three independent biological replicates were measured with all methods described below.

For metatranscriptome analysis, 2 mL LifeGuard Soil Preservation Solution (MO BIO Laboratories, Carlsbad, CA) was added to 1 mL concentrated filaments. Filaments were cleaned from debris mechanically using a stereo microscope. For the metabolite profile, 2.5 mL sample was frozen in liquid nitrogen.

### *Assessment of photosynthetic activity*

The rETR of the top and bottom layers were measured in triplicates with a pulse‐amplitude modulated fluorometer (PAM 2500; Heinz Walz GmbH, Effeltrich, Germany) as previously described (Herburger *et al*., 2015). The light response curves were fitted according to Webb *et al*. (1974) assuming photoinhibition.

### *Light and transmission electron microscopy*

Light microscopy was conducted according to Pichrtová *et al*. (2018) at an Olympus BX51 light microscope (Nomarski differential contrast, phase contrast) with Olympus Camedia C‐5060Z (Olympus, Tokyo, Japan). For transmission electron microscopy, vegetative field samples were chemically fixed according to Holzinger *et al*. (2009). The whole procedure, including ethanol dehydration, embedding in modified Suprr's resin (Low viscosity embedding kit, Science Services, Munich, Germany), was conducted immediately upon sampling. Ultrathin sections were prepared from the embedded material (Reichert Ultracut, Leica Mikrosysteme Handelsges.m.b.H., Wien, Austria), counterstained and investigated with a Libra® 120 TEM (Carl Zeiss AG, Oberkochen, Germany) at 80 kV and images were recorded with a 2 k SSCCD camera (Albert Tröndle Restlichtverstärker Systeme, Moorenweis, Germany).

### *Metatranscriptomics*

Total RNA was extracted using the CTAB protocol as described by Rippin *et al*. (2016). Genomic DNA was removed by incubating the solution with DNase I Thermo Scientific (Waltham, MA) and subsequently purifying it using the RNeasy MinElute Cleanup kit (Qiagen, Hilden, Germany) according to manufacturer's instructions. The resulting samples and an additional mix of all replicates (reference) were subjected to mRNA enrichment using oligo‐(dT) beads, fragmented and reverse‐transcribed into cDNA. After adapter ligation, the sample libraries were sequenced on an Illumina HiSeq 2500 (2 × 125 bp), operated with the HiSeq Control Software 2.2.38 and RTA 1.18.61, and the normalized reference library was sequenced on an Illumina MiSeq (2 × 300 bp) using the MiSeq Control Software 2.5.0.5 and RTA 1.18.54. The base call files were converted to fastq using bcl2fastq‐1.8.4. All raw reads were uploaded to SRA and are accessible via the bioproject PRJNA498913.

Prior to assembly, the raw reads of the reference were trimmed using Trimmomatic 0.35 (Bolger *et al*., 2014), filtered using SortMeRNA 2.1 (Kopylova *et al*., 2012) with the SILVA SSU NR Ref 119 and LSU Ref 119 database (Quast *et al*., 2013) and PrinSeq Lite 0.20.4 (Schmieder and Edwards, 2011) as well as combined, in case paired‐end reads were overlapping, using COPE 1.2.5 (Liu *et al*., 2012). The remaining reads were assembled to contigs using Trinity 2.0.6 (Grabherr *et al*., 2011) and the assembly was subjected to quality analysis conducted with scripts from the Trinity package and BUSCO 3.0.2 in combination with the embryophyta database (Simão *et al*., 2015). SSU rRNA gene reads, filtered out by SortMeRNA, were fed into EMIRGE 0.61.0 (Miller *et al*., 2011) to assess community structure of our samples.

The assembled contigs were annotated using the Trinotate pipeline 3.0.0 (http://trinotate.github.io/), including TransDecoder 2.1 (http://transdecoder.github.io/), NCBI BLAST+ 2.3.0 (Altschul *et al*., 1990), HMMER 3.1 b (Finn *et al*., 2011), SignalP 4.1 (Petersen *et al*., 2011), TMHMM 2.0 c (Krogh *et al*., 2001), RNAmmer 1.2 (Lagesen *et al*., 2007) and the databases SWISSPROT (Bairoch and Apweiler, 1997), PFAM 3.1b2 (Sonnhammer *et al*., 1997), Phytozome 12 (*M*. *polymorpha*, *Physcomitrella patens*), 1KP (*Coleochaete irregularis*, *Cosmarium ochthodes*, *Cylindrocystis brebissonii*, *Euastrum affine*, *Mesotaenium braunii*, *Mougeoutia* sp., *Penium margaritaceum*, *Straurastrum sebaldi*, *Zygnema* sp., *Zygnemopsis* sp.; Matasci *et al*., 2014), the transcriptomes of *Mesostigma viride* (https://dx.doi.org/10.6084/m9.figshare.1604778) and *Z*. *circumcarinatum* (Rippin *et al*., 2017) as well as the genomes of *Chara braunii* (Nishiyama *et al*., 2018) and *Klebsormidium nitens* (Hori *et al*., 2014).

To identify differentially expressed genes, sample raw reads were mapped onto the assembly with Bowtie 1.1.2 (Langmead *et al*., 2009), transcript abundance was estimated with RSEM 1.2.30 (Li and Dewey, 2011) and differentially regulated contigs were detected with edgeR (Robinson *et al*., 2010). Genes with a corrected *p*‐value (Benjamini and Hochberg, 1995) of less than 0.001 and a fold change of at least 4 were considered differentially expressed. Gene set enrichment analyses were performed using GoSeq 1.26.0 for GO terms (Young *et al*., 2010) and clusterProfiler 3.2.11 (Yu *et al*., 2012) for KEGG annotations. The false discovery rate threshold for significance was set to 0.05. GO network data were modified and retrieved with the online tool REVIGO (Supek *et al*., 2011).

### *GC–MS‐based metabolite profiling*

Chemical derivatization and GC–MS metabolite profiling analysis were performed according to Fiehn *et al*. (2008). Freeze‐dried samples were homogenized using a ball mill for 30 s at 20 s^−1^ (TissueLyser II, Qiagen, Düsseldorf, Germany). Then, 10 mg of each homogenate was suspended in 1 mL ice‐cold (−20 °C) water:acetonitrile:isopropanol (2:3:3) containing 4 μg mL^−1 13^C_6_‐Sorbitol (Campro Scientific GmbH, Berlin, Germany) and extracted for 10 min at 4 °C with continuous shaking at 1400 rpm (Compact Digital Microplate Shaker, Thermo Scientific). Insoluble material was removed by centrifugation at 20 000*g* for 5 min. A volume of 25 μL of the supernatant was collected and dried for 3 h in a vacuum centrifuge (Savant SPD111V P2 SpeedVac kit, Thermo Scientific). The same steps were performed on a blank sample for quality control. Vacuum‐dried samples were re‐suspended in 10 μL of pyridine (Sigma‐Aldrich, St Louis, USA) amended with 20 mg mL^−1^ methoxyamine‐hydrochloride (Sigma‐Aldrich) and incubated at 28 °C for 90 min, with continuous shaking in a thermomixer (Ditabis® MHR 13, GML, Innsbruck, Austria). Ninety microliters of *N*‐methyl‐*N*‐trimethylsilyl‐trifluoroacetamide (Aldrich 394,866–10 × 1 mL, Sigma‐Aldrich) were then added and the reaction continued for 30 min at 37 °C. After cooling, the content of each tube was transferred to a 2 mL clear glass autosampler vial with micro insert (Agilent Technologies, Santa Clara, CA) for injection. Samples were injected between 2 and 24 h after derivatization.

Starting 2 h after derivatization, 1 μ of each sample was injected using a TriPlus RSH autosampler on a Trace 1300 gas chromatograph coupled to a TSQ8000 triple quadrupole mass spectrometer and operated with the Xcalibur software (Thermo Scientific). Before and after each injection, the syringe was washed three times with 5‐μL hexane and three times with 5 μL ethyl acetate. The injector was operated in splitless mode, opening the split vent after 4 min, with a constant flow of helium at 1 mL min^−1^ and at a constant injector temperature of 250 °C. The glass liner (#23467, Restek, Bellefonte, USA) was changed before each series of 25 sample injections. A 30 m long, 0.25 mm internal diameter Rxi‐5Sil MS from Restek with 0.25 μm Crossbond 1,4‐bis(dimethylsiloxy)phenylene dimethyl polysiloxane film and an additional 10 m integrated guard column was used (#13623–127, Restek). The oven temperature was held at 70 °C for 7 min then ramped at 10 °C min^−1^ to 330 °C, and held constant for 7 min. The transfer line temperature between the gas chromatograph and mass spectrometer was set to 300 °C. Electron impact ionization was employed at 70 eV with an ion source temperature of 330 °C. A mix of alkanes dissolved at 2 mg L^−1^ in hexane was injected in the middle of the queue to allow for the conversion of retention times into Kováts' alkane‐based retention indices (Kováts, 1958). Mass spectra were acquired in full scan mode from *m/z* 50 to 600 at 5 spectra per second, and raw data files were analysed with the ‘Automated Mass‐spectral Deconvolution and Identification System’ (AMDIS) v2.71 software (Stein, 1999). Deconvoluted mass spectra and associated retention indexes were then compared against a custom‐built mass spectral library and the National Institute of Standards and Technology (NIST, Gaithersburg, MD), Golm, and Fiehn databases (Kopka *et al*., 2005; Kind *et al*., 2009), using AMDIS and the NIST MS Search v2.0 program.

Identifications were only considered valid given a match of the spectrum (match score > 80 in AMDIS, or above 800 in MS search) and retention index (±3 U difference from the in‐house library) with library data. The most prominent unidentified compounds are reported as unknowns with their retention index and a characteristic fragment. A specific fragment was selected for the relative quantification of each compound based on the data generated with AMDIS and the corresponding peak areas were then determined at the expected compound retention times using the Xcalibur v2.2 processing software (Thermo Scientific) with the *genesis* algorithm. All peak integrations were subsequently assessed using the Xcalibur Quan browser. Missing values were replaced with the manually integrated background level at the expected peak retention time. Relative values of metabolite contents were determined by normalizing the peak areas of each metabolite to that of the internal ^13^C_6_‐Sorbitol standard and to the sample dry weights.

Statistical evaluation of the data was performed with *R* (R Core Team (2019). The data were scaled by setting the highest value to one and adjusting the other values accordingly. After performing a principal component analysis, one replicate of sample from the bottom of the layer was removed (Fig. S3). The remaining replicates of top and bottom layers were tested for differences using the Brown–Forsythe test (Brown and Forsythe, 1974). Adjusted *p*‐values were corrected according to Benjamini and Hochberg (1995).

## Supporting information


**Fig S1.** rETR curves of top and bottom layer. Top: ETRmax = 29.8; *α* = 0:325; *β* = −0:003; I_K_ = 91.5. Bottom: ETRmax = 46.7; *α* = 0:299; *β* = −0:01; I_K_ = 156.3.

**Table S1.** Supporting Information

**Table S2.** Supporting Information

**Table S3.** Supporting Information

**Table S4.** Supporting Information
