# Supplementary material for: Metatranscriptomic and metabolite profiling reveals vertical heterogeneity within a Zygnema green algal mat from Svalbard (High Arctic)
Source: Environ Microbiol. 2019 Sep 11;21(11):4283–99. doi: 10.1111/1462-2920.14788 (PMC6899726; doi:10.1111/1462-2920.14788)
Supplement: Supplementary file 1 — Fig S1. rETR curves of top and bottom layer. Top: ETRmax = 29.8; α = 0:325; β = −0:003; IK = 91.5. Bottom: ETRmax = 46.7; α = 0:299; β = −0:01; IK = 156.3. [file EMI-21-4283-s001.pdf]

Supporting Information

Metatranscriptomic and metabolite profiling reveals vertical heterogeneity within a *Zygnema* mat from Longyearbyen, Svalbard

Martin Rippin[1 3], Martina Pichrtová[2], Erwann Arc[3], Ilse Kranner[3], Burkhard Becker[2], Andreas Holzinger[3]

[1]University of Cologne, Botanical Institute, Cologne, Germany
[2]Charles University, Department of Botany, Prague, Czech Republic
[3]University of Innsbruck, Department of Botany, Innsbruck, Austria

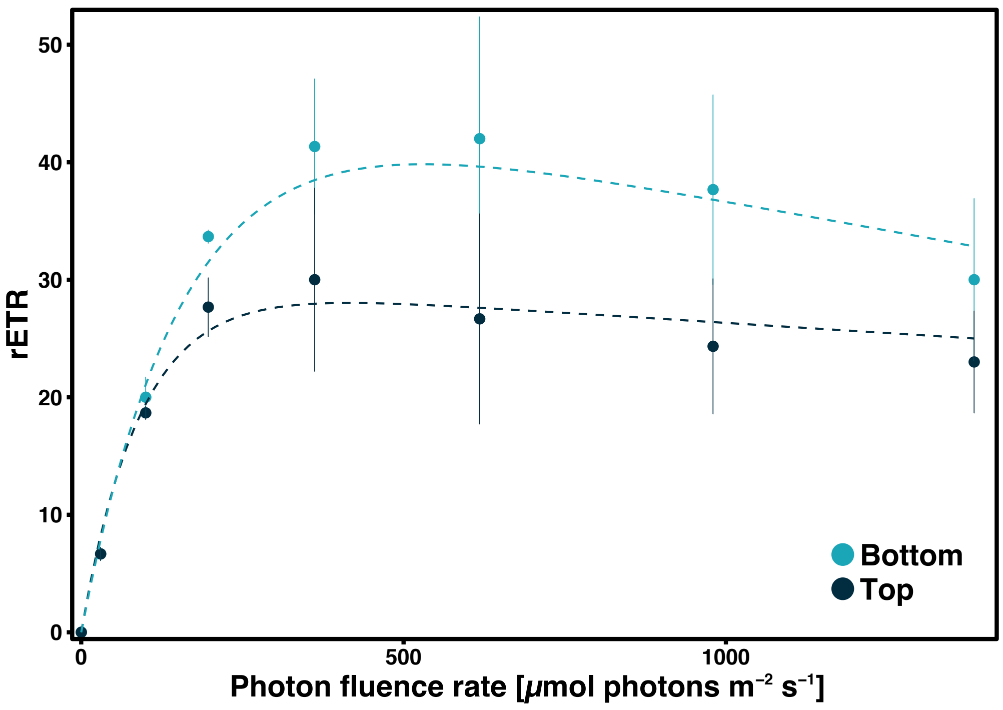

Fig. S1. rETR curves of top and bottom layer. Top: ETR_{max} = 29.8; $\alpha = 0.325$; $\beta = -0.003$; I_K = 91.5. Bottom: ETR_{max} = 46.7; $\alpha = 0.299$; $\beta = -0.01$; I_K = 156.3.

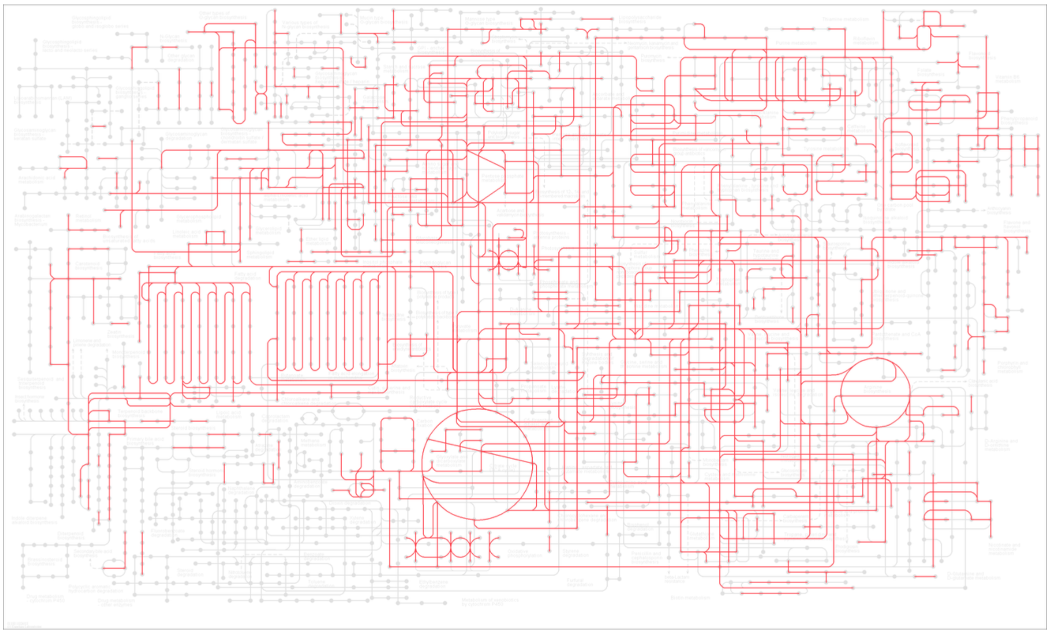

Fig. S2. Mapping of KEGG ath annotations on the metabolic pathway map ko01100.

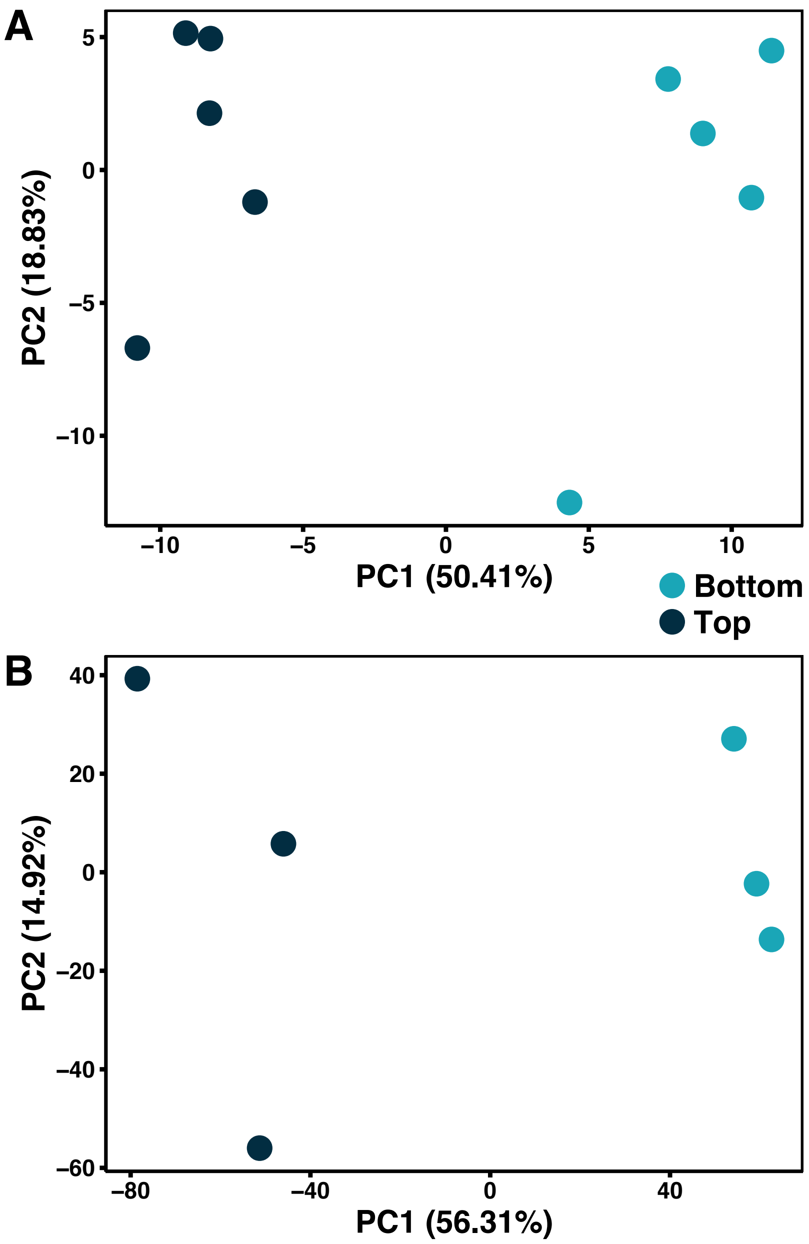

Fig. S3. Principal component analysis of the top and bottom layer replicates. A: Metabolite Profiling. B: Metatranscriptome.
